# The safety and efficacy of fibrinogen and thrombin-based collagen fleece for treatment of pleural injury

**DOI:** 10.1186/1749-8090-10-S1-A151

**Published:** 2015-12-16

**Authors:** Norifumi Tsubokawa, Yoshihiro Miyata, Takahiro Mimae, Shinsuke Sasada, Tomoharu Yoshiya, Masaoki Ito, Yasuhiro Tsutani, Takeshi Mimura, Morihito Okada

**Affiliations:** 1 Department of Respiratory Surgery, National Hospital Organization Kure Medical Centre and Chugoku Cancer Centre, Kure, Hiroshima, 737-0023, Japan; 2 Department of Surgical Oncology, Hiroshima University, Hiroshima, 734-8551, Japan

## Background/Introduction

Fibrinogen/thrombin-based collagen fleece (TachoSil) can be applied to repair pleural injury, however, little is known about the impact of its use on lung.

## Aims/Objectives

This study evaluated the long-term histologic changes associated with the use of TachoSil to repair pleural injury in a canine model.

## Method

Three or four visceral pleural defects of 10 × 10 mm were created in one animal via left thoracotomy. After air leakage was confirmed, each pleural defect was covered with Tachosil. Animals were sacrificed on postoperative days (POD) 0, 4, 7, 14, 28, and 56, and the repair sites were histologically evaluated on each postoperative days.

## Results

A total of 20 TachoSil were applied to pleural defects. All animals survived thorough the observational period. No pneumothorax was found and two adhesions (10%) were observed on the pleura covered by TachoSil at the time of sacrifice. Tachosil was macroscopically detectable until POD 28 and only macroscopically detectable on POD 56. Histologically, inflammatory cells infiltrated the TachoSil from the pleural defect, and pleural mesothelium comprised the regenerated outermost layer of the TachoSil until POD 4. Inflammatory cells, myofibroblasts and neovascular vessels then spread over the entire TachoSil. The number of inflammatory cells decreased, and then myofibroblast and neovascular vessels replaced the entire TachoSil, which was not left as foreign material. In addition, the elastic layer started to regenerate from both edges and completely repaired the pleural defect. Importantly, the lung parenchyma was not influenced because these healing processes occurred only inside the TachoSil.

## Discussion/Conclusion

TachoSil safely and effectively repaired the pleural injury without affecting lung parenchyma, and then became biodegraded over the long term.

